# Leukocytoclastic Vasculitis Secondary to Anti-Tumor Necrosis Factor Therapy in Inflammatory Bowel Diseases: A Multicenter Retrospective Cohort Study

**DOI:** 10.3390/jcm12093165

**Published:** 2023-04-28

**Authors:** Rogério Serafim Parra, Júlio Maria Fonseca Chebli, Liliana Andrade Chebli, Sérgio Figueiredo de Lima Junior, Manoel Alvaro Lins Neto, Terry Rocha de Medeiros, Francesca Maia Faria, Marley Ribeiro Feitosa, Cintia Maura Caseiro Nigro, Omar Féres

**Affiliations:** 1 Department of Surgery and Anatomy, Ribeirão Preto Medical School, University of São Paulo, Ribeirão Preto 14048-900, Brazil; 2 Division of Gastroenterology, Department of Medicine, Inflammatory Bowel Disease Center, Federal University of Juiz de Fora, Juiz de Fora 36036-900, Brazil; 3 General and Digestive Surgery Service, João de Barros Barreto University Hospital, Belém 66073-000, Brazil; 4 Department of Gastrointestinal Surgery, Federal University of Alagoas—UFAL, Maceió 57051-090, Brazil; 5 Hospital Professor Edmundo Vasconcelos, São Paulo 04038-905, Brazil; 6 Department of Pathology and Forensic Medicine, School of Medicine of Ribeirão Preto, University of São Paulo, Ribeirão Preto 14048-900, Brazil

**Keywords:** biologic therapy, Crohn’s disease, inflammatory bowel disease, ulcerative colitis, vasculitis

## Abstract

Background: Vasculitis is an uncommon complication of biologics used to treat inflammatory bowel disease (IBD). This study describes a case series of vasculitis induced by anti-tumor necrosis factor (TNF) therapy in IBD patients. Methods: Retrospective assessments were performed using the medical records of adult IBD patients who underwent outpatient clinical follow-ups between January 2010 and December 2019 in order to identify patients with vasculitis caused by anti-TNF therapy. Results: There were 2442 patients altogether. Of these, 862 (35%) took anti-TNF medication. Five patients (0.6% of the overall patients; *n* = 3 (60%) Crohn’s disease; *n* = 2 (40%), ulcerative colitis) were identified as having leukocytoclastic vasculitis (LCV) due to anti-TNF therapy; these patients were white, female, and non-smokers. The mean age of LCV diagnosis was 32.2 years, and the mean IBD duration was 7.2 years. The mean time between the start of biologic therapy and LCV onset was 30.8 months. Most of the patients were using adalimumab (80%; *n* = 4). All the patients were in remission at the time of the LCV diagnosis, and the vasculitis affected the skin in all cases. Anti-TNF therapy was discontinued in the five abovementioned patients, and the response of LCV to the oral steroids was significantly positive. Remarkably, all five patients experienced complete remission from LCV within 4–12 weeks after starting prednisone therapy, and none of them had LCV recurrence in the follow-up period (a mean duration of 28 months). Conclusions: LCV is an unusual complication of anti-TNF therapy in the IBD setting. In this context, clinicians should have a high degree of suspicion of LCV in patients who develop an unexplained cutaneous rash.

## 1. Background

Anti-tumor necrosis factor (anti-TNF) therapy has been successfully used for more than 20 years to treat inflammatory bowel diseases (IBDs), such as Crohn’s disease (CD) and ulcerative colitis (UC) [1]. However, these drugs may be associated with several side effects, many of which include immune-mediated reactions, such as paradoxical psoriasis, vasculitis, and lupus-like syndromes [2,3,4,5,6].

Vasculitis resulting from drug use includes a wide variety of clinical and pathological conditions. Vasculitis can be associated with systemic disease or probable etiology (e.g., rheumatoid vasculitis, lupus vasculitis, etc.). Moreover, vasculitis associated with a potential etiology should have a prefix specifying the association (e.g., hepatitis-B-virus-associated vasculitis) [7]. Some therapeutic agents, including biologics, can induce a variety of vasculitic manifestations, ranging from small-vessel hypersensitivity vasculitis to leukocytoclastic vasculitis (LCV) [8]. The pathogenic mechanisms remain to be defined and appear to be multifactorial.

Vasculitis is an unusual complication that is induced by TNF antagonists [3,9,10,11,12,13,14]. Ramos-Casals et al. [9] identified 33 cases of vasculitis in patients following the initiation of TNF antagonists to treat rheumatologic diseases or IBD via a Medline search of articles published between January 1990 and December 2006. In their report, only seven patients had IBD [9]. In a recent meta-analysis of 48 studies on 29,776 patients treated with anti-TNF medications for IBD, the authors demonstrated that the incidence of dermatological events in patients with IBD on anti-TNF medications was high [15]. The most common adverse event was psoriasis or psoriasiform, followed by the development of rashes, eczema, and skin infections. No cases of cutaneous vasculitis were reported [15].

At the start, the lesions present as localized erythema and macular purpura or urticarial papules that progress to palpable purpura, usually bilateral and symmetrical and frequently involving the lower limbs [16]. Although vasculitis induced by anti-TNF therapy primarily affects the skin, related multiorgan involvement has been reported in a quarter of all patients [3,9]. In this scenario, life-threatening complications can occur, which indicates the potential severity of this disease. Thus, there is an urgent need to evaluate the clinical features, the outcomes, and the management of vasculitis attributed to the use of anti-TNF agents in the broad IBD patient population. Thus, the present study was conducted to assess the clinical features, the histologic findings, and the outcomes of patients with IBD who were also found to have anti-TNF-α-therapy-induced vasculitis.

## 2. Methods

### 2.1. Study Type and Population

In this multicenter retrospective cohort study, the electronic medical records of 2242 adult IBD patients undergoing outpatient clinical follow-up between January 2010 and December 2019 at five IBD tertiary centers in Brazil were retrospectively assessed. The primary study objective was to identify patients with IBD-associated vasculitis. Subsequently, researchers at each IBD center reviewed the medical records in detail to identify patients with vasculitis specifically induced by anti-TNF treatment as a secondary objective.

To ensure a virtually complete identification of the cases, we used different supplementary data retrieval sources. First, the patients’ electronic records were retrospectively retrieved from the databases of the participants’ tertiary care medical centers using the International Classification of Disease (ICD-9 and ICD-10) codes for inflammatory bowel disease, including Crohn’s disease (CD) and ulcerative colitis (UC). Second, we used the computerized databases of the IBD units of the participating hospitals, which store standardized reports of all IBD-associated complications/conditions, to identify all biopsy-positive cases of IBD-associated vasculitis/vasculitides. Finally, the electronic records of the patients with IBD who had concomitant biopsy-proven vasculitis/vasculitides were reviewed in detail by each of the researchers at the centers involved in the study.

A diagnosis of anti-TNF-therapy-induced vasculitis was made for the patients who presented with one or more clinical manifestations of vasculitis (e.g., systemic or skin involvement) during anti-TNF therapy. This diagnosis was confirmed by the findings of a histopathology report following a biopsy performed on at least one site of involvement and by the absence of other likely causes of vasculitis, such as an infection, the concurrent use of other medications to treat conditions other than IBD that may be associated with vasculitis, concomitant rheumatic and non-IBD autoimmune disease, and malignancy [3]. A diagnosis of cutaneous vasculitis affecting small- and medium-sized vessels in the skin and subcutaneous tissue was made primarily through biopsy and an examination of hematoxylin- and eosin-stained sections, as described in the literature [17]. An immediate diagnosis of vasculitis was made if inflammatory infiltrates were identified within and around the vessel walls, accompanied by fibrin deposition [18,19]. In addition, a diagnosis of LCV was confirmed with a punch biopsy showing (1) a superficial perivascular inflammatory infiltrate; (2) the predominance of neutrophils in the capillary walls and postcapillary venules; (3) leukocytoclasis (the fragmentation of neutrophils into scattered nuclear fragments secondary to karyorrhexis); and (4) the perivascular and interstitial extravasation of erythrocytes [18,19].

The complete remission of LCV was defined as the absence of residual active cutaneous lesions, with the occasional presence of skin scarring during follow-up. Patients who presented with any other known cause of vasculitis, such as infections, or those who used other medications (i.e., non-anti-TNF agents) to treat conditions other than IBD (i.e., rheumatic and autoimmune diseases, such as rheumatoid arthritis, Sjogren’s syndrome, Behçet’s disease, systemic lupus erythematosus, relapsing polychondritis, and malignancies, except for skin cancer) and associated systemic vasculitis, such as primary small-vessel vasculitis (ANCA-associated vasculitis, cryoglobulinemic vasculitis, and IgA vasculitis) were excluded from this analysis.

### 2.2. Measurements and Outcomes

The following relevant patient data were extracted from the medical records of the patients diagnosed with vasculitis secondary to anti-TNF therapy by researchers at each IBD center: (1) the patient’s characteristics (i.e., age, gender, and smoking status); (2) the variables related to the disease (e.g., the IBD type, the disease duration and location according to the Montreal classification [20], IBD therapy, and IBD activity at the time of the vasculitis diagnosis); (3) the anti-TNF therapy (e.g., the type of anti-TNF agent, the duration of therapy, and the time interval between the initiation of the anti-TNF agent and a diagnosis of vasculitis); (4) vasculitis-related data (e.g., the clinical features, including organ involvement, the laboratory findings (including those obtained from an autoimmune autoantibody panel), the histologic characteristics, the type of vasculitis, the response to therapy, the outcome after cessation of the anti-TNF agent that induced the vasculitis, and subsequent IBD therapy); and (5) information pertaining to the risk of anti-TNF-induced vasculitis.

In terms of the instruments used in the study, CD clinical activity was measured using the Harvey–Bradshaw Index (HBI). A HBI score of ≤4 indicated clinical remission, and a score of ≥5 indicated active disease. The disease activity of the UC patients was determined according to the guidelines of the European Crohn’s and Colitis Organisation [21]. Endoscopic activity was evaluated via ileocolonoscopy performed within three months after a diagnosis of vasculitis. An endoscopic Mayo Score of 0 or 1 for UC and the absence of ulcers or erosions for CD were the endoscopic criteria for remission.

### 2.3. Statistical Analysis

A descriptive statistical analysis was conducted using frequency, percentage, mean, and range to describe the variables. Fisher’s exact test was used to evaluate the nominal variables. The statistical analysis was performed using GraphPad InStat^®^ 3.05.

### 2.4. Ethical Considerations

The present study was approved by the Ethics Committee for Research in Humans (approval number 05859218.3.0000.5440, #3.117.993). The patients included in the study consented to a review of their medical records for research purposes by providing informed written consent to the publication of a case series and to any other related publications. The procedures were performed in accordance with the ethical standards of the committee responsible for human experimentation (institutional and national) and the later amendments of the World Medical Association Declaration of Helsinki (1964). 

## 3. Results

The medical records of the patients with CD or UC (*n* = 2442) were evaluated. Of these, 862 (35%) took anti-TNF medications. Five cases (0.6% of the patients on anti-TNF medication) of biopsy-proven vasculitis secondary to anti-TNF therapy were identified, all of which were LCV. Two IBD patients with vasculitis were excluded from this research because they had other conditions that could be associated with vasculitis (i.e., hepatitis C virus (*n* = 1) and rheumatoid arthritis (*n* = 1)).

All the patients were female, white, and non-smokers. Three of these patients (60%) had CD, and the other two (40%) had UC. The mean age at LCV diagnosis was 32.2 years (range: 20–38 years), and the mean age at IBD diagnosis was 25 years (range: 18–36 years). The mean disease duration was 7.2 years (range: 1–19 years). The mean time between the start of biologic therapy and a diagnosis of LCV was 30.8 months (range: 12–60 months). Four of the five patients used adalimumab (80%), and the remaining patients used infliximab (20%). None of the patients presented with any extraintestinal manifestations. The demographic and clinical data of the patients are provided in Table 1.

All the patients were in deep remission (i.e., endoscopic and clinical remission) at the time of their LCV diagnosis. The fecal calprotectin levels were ≤250 µg/g in three of the patients with LCV. Fecal calprotectin levels were not available for the other two patients with LCV. The two patients with UC presented with an endoscopic Mayo Score of 0. The CD patients exhibited complete mucosal healing when the endoscopic evaluations were performed in the period between the time that the skin lesions appeared and three months after a diagnosis of vasculitis was made. 

### 3.1. Clinical, Laboratory, and Histologic Characteristics of Anti-TNF-Therapy-Induced Vasculitis

**Figure 1:**
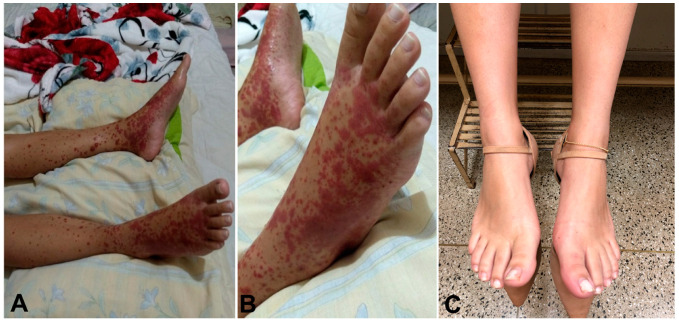
Patient with Crohn’s disease on adalimumab therapy presenting multiple and confluent erythematous macules and palpable purpura in a symmetrical distribution involving both lower legs and feet (**A**,**B**). Complete recovery after two months on steroids (**C**).

**Figure 2:**
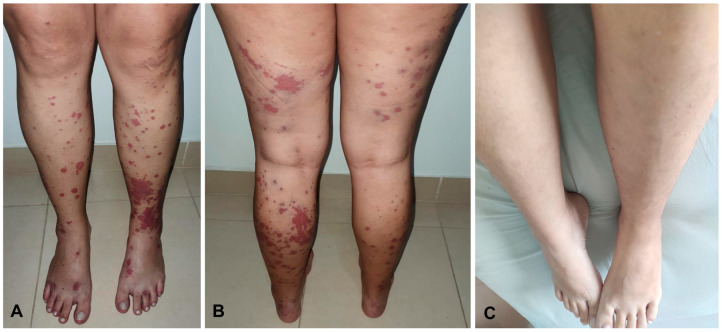
Patient with extensive ulcerative colitis in deep remission on adalimumab, presenting with multiple and confluent erythematous macules and palpable purpura in a symmetrical distribution on both lower legs and feet (**A**,**B**). Twelve weeks later, the complete resolution of the skin lesions after the initiation of corticosteroids therapy could be seen (**C**).

**Figure 3:**
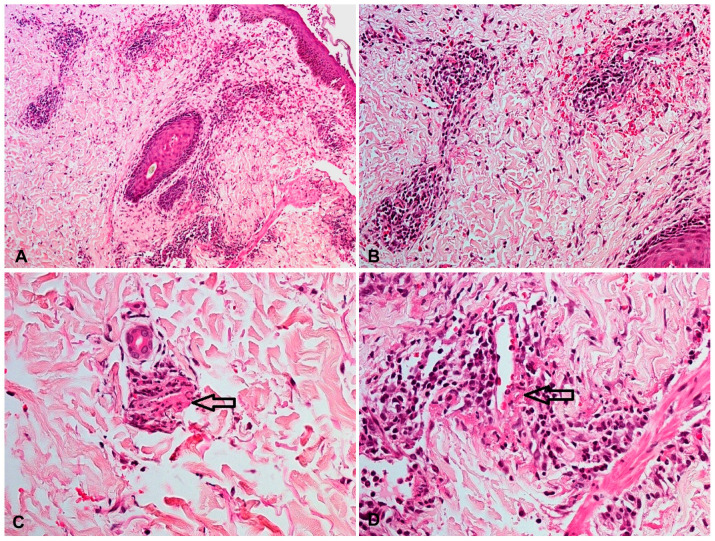
H&E ((**A**), 100×) ((**B**), 200×) staining of skin lesions showing a perivascular inflammatory pattern upon dermis and erythrocyte extravasation. Perivascular neutrophilic inflammatory infiltration, neutrophil degeneration forming nuclear dust, and the fibrinoid necrosis of the vessels with fibrin extravasation are consistent findings with leukocytoclastic vasculitis (**C**,**D**).

Vasculitis affected the skin in all five patients: four of the patients presented with palpable purpura, three had painful ulcerated lesions, and one had erythematous macules (Figure 1 and Figure 2). In all the patients, the lower extremity was, bilaterally involved. One patient also presented lesions in the upper extremity and the trunk, in addition to the lower extremity involvement. None of the patients exhibited extracutaneous manifestations or had any systemic symptoms, such as fatigue, fever, or arthralgia. Notably, all five skin biopsy specimens showed LCV upon histologic examination (Figure 3).

The laboratory investigations of the five patients, which included a platelet count, a C-reactive protein analysis, a urinalysis, a creatinine analysis, and a hepatic function panel, were unremarkable. The test results for human immunodeficiency virus (HIV) 1, HIV-2, Epstein–Barr virus, cytomegalovirus serology, hepatitis B surface antigen, immunoglobin (Ig)M antibody to hepatitis B core antigen, anti-hepatitis A virus IgM, and anti-hepatitis C virus were all negative. The serum levels of IgG, IgA, and IgG4 and complement (C)3 and C4 were all within their normal ranges. In addition, the five patients tested negative for rheumatoid factor, anticardiolipin antibodies, cryoglobulins, perinuclear antineutrophil cytoplasmic antibodies, antinuclear antibodies (anti-Ro), antiphospholipid antibodies, anti-double-stranded DNA antibodies, anti-Smith antibodies, anti-nuclear ribonucleoprotein antibodies, and anti-Scleroderma-70 antibodies.

### 3.2. Treatment of Leukocytoclastic Vasculitis and Patient Outcomes

Anti-TNF therapy was discontinued in the five patients, all of whom experienced complete remission from skin lesions between 4 and 12 weeks (mean of 8 weeks) after starting prednisone (0.5 mg/kg/day). The average duration of steroid therapy was 3 months (range: 2.5 to 5.0 months). To maintain the treatment for IBD, the biologic mechanism of action was changed for three patients (the two CD patients switched to ustekinumab, and one patient with UC switched to vedolizumab). Two of the five patients were re-challenged with another anti-TNF agent (i.e., infliximab) after the discontinuation of adalimumab. None of the patients experienced vasculitis recurrence after switching their biologic therapy (a mean follow-up of 21.4 months, range: 12–28 months).

## 4. Discussion

Five patients with IBD who were in remission at the time the study was conducted and who presented with LCV as a rare paradoxical complication of anti-TNF therapy were evaluated in this research. There was evidence that anti-TNF therapy was a strong risk factor for LCV. Palpable purpura was the most common clinical finding. The five patients experienced the complete resolution of cutaneous lesions after discontinuing the use of the anti-TNF agent and starting therapy with a short course of oral prednisone. In addition, none of the patients experienced vasculitis recurrence after switching to another agent within the same class or changing to a biologic agent with a non-TNF-related mechanism of action. To the best of our knowledge, this is one of the first multicenter case series to describe LCV secondary to anti-TNF therapy in a broader cohort formed exclusively of IBD patients.

The treatment of IBD has changed dramatically since the introduction of anti-TNF therapy [1]. However, safety issues involving the anti-TNF agents as a class are one of the main concerns. For example, the incidence of dermatological events among patients with IBD who have been treated with anti-TNF medications is high. In addition, TNF blockers have been associated with the development of autoimmune diseases, such as paradoxical psoriasis, demyelinating central nervous system disease, vasculitis, and lupus-like syndrome [9,15]. As observed in our case series, LCV is the most frequent type of anti-TNF-therapy-induced vasculitis, and it predominates in women [22]. In addition, the typical clinical features of cutaneous involvement of anti-TNF-therapy-induced LCV, including symmetric erythematous macules or ulcerated and palpable purpuric lesions, were present in our patients. Most of the patients in the current study had palpable purpura (four of the five). Indeed, palpable purpura is the most frequent clinical manifestation of LCV associated with anti-TNF therapy [9,22].

The pathogenesis of LCV is unknown, although it is understood that the development of antibodies against TNF blockers can lead to immune-complex-mediated hypersensitivity vasculitis [3]. It has also been hypothesized that the blockade of the TNF-α signaling pathway induces a cytokine imbalance, with a shift from a T helper 1 pattern to a T helper 2 pattern, which renders the patient susceptible to vasculitis [23]. It seems that LCV can occur during disease quiescence or active IBD. Interestingly, in the current case series, the five IBD patients were in remission at the time that the LCV diagnosis was made. LCV can be triggered by medications, infections, surgery, chemicals, and other autoimmune diseases [9,24,25,26]. Although cutaneous vasculitis may be an uncommon extraintestinal manifestation of IBD, especially CD, this condition is typically reported at the onset of symptoms or during periods of disease activity. In our case series, all the patients were in deep remission (i.e., endoscopic and clinical remission) at the time of their LCV diagnosis [27]. Additionally, the complete remission of skin lesions occurred a mean of eight weeks after the withdrawal of the anti-TNF therapy and the initiation of systemic steroids. These data strongly support our hypothesis that the cases of LCV in our case series were due to anti-TNF therapy and not IBD itself.

In all five patients, the results of detailed laboratory investigations for inflammatory markers, autoimmune markers, immunoglobulin (IgG, IgA, and IgG4) and complement (C3 and C4) serum levels, viral serology, infectious work-up, urinalysis, creatinine, and liver function were unremarkable. Although a patient’s clinical history and a physical examination and laboratory work-up are important for a differential diagnosis of LCV, a histology is essential to confirm a diagnosis of vasculitis and to avoid a delayed or erroneous diagnosis that could lead to improper management [19,28]. The differential diagnosis includes systemic primary small-vessel vasculitis (such as ANCA-associated vasculitis, cryoglobulinemic vasculitis, and IgA vasculitis), thrombocytopenic purpura, infections, and rheumatic and autoimmune diseases, such as rheumatoid arthritis, Sjogren’s syndrome, Behçet’s disease, systemic lupus erythematosus, and relapsing polychondritis [7]. As previously mentioned, the detailed clinical data and laboratory investigations into these conditions were unremarkable. Lastly, a skin biopsy can easily differentiate between benign pigmented purpura and LCV, given the lack of any obvious features of vasculitis, such as fibrinoid necrosis and vessel wall disruption [19].

Vasculitis in IBD patients remains a rare and, fortunately, mild complication for most patients, and it mainly relates to the drugs used to treat IBD [29,30,31]. In a retrospective review of the patients evaluated at the Mayo Clinic from January 1998 to March 2011, only eight patients with vasculitis associated with anti-TNF therapy were identified, and, of these, only four had IBD [3]. In our case series, the prevalence of LCV was less than 1% (i.e., 0.6%) of the IBD patients who were using anti-TNF medications. This is in accordance with the findings of another study that reported 7 cases (1%) of vasculitis among 732 patients treated with anti-TNF agents for various conditions [32].

A recently published study performed a systematic review synthesizing the data on LCV in patients with UC [33]. This study represents the largest case series (*n* = 22) published on the topic. The etiology of LCV was UC irrespective of disease activity in most of the patients (95.5%). However, the therapeutic drug (infliximab) was identified as the trigger factor for LCV in only one patient (4.5%). As presented in our case series, rashes were most commonly bilateral and manifested on the lower extremities (72.7%). Localized pain was present in about 40% of the cases, as observed in our study. Only 18% of the patients with UC were in remission at the time that the rash appeared.

In about 30% of patients with CD, the rash spreads upwards to include the trunk and upper extremities, as described in our case series [5]. The exact incidence of LCV in patients with IBD is unknown, and the association/concomitance between the two conditions has important implications for the management of IBD. This allows our study to contribute to the current knowledge on this topic. In our study, all the patients were in deep remission from IBD. A previous study found that the majority of the patients developed LCV as a reactive manifestation of IBD [33]. However, in our series, all the patients had IBD in deep remission, and the other well-known causes of vasculitis were excluded.

LCV appears to be a paradoxical complication of the prolonged use of anti-TNF therapy. In fact, in the present series, the mean time between the start of the anti-TNF agent and an LCV diagnosis was 30.8 months. This finding is in accordance with that found in the retrospective review performed by Sokumbi et al. [3] at the Mayo Clinic in Rochester, Minnesota, which reported a mean duration of 34.5 months between the initiation of anti-TNF therapy and the development of vasculitis. 

In cases of LCV where the involvement is confined to the skin, the discontinuation of the anti-TNF agent, along with a short course of oral steroids, is usually sufficient to resolve the vasculitis within a couple of months [23]. Indeed, in our case series, the five patients responded favorably to the withdrawal of the TNF antagonist and to the initiation of a brief course (a mean of eight weeks) of prednisone. It is important to mention that there have been cases where aggressive immunosuppressive therapy has been preferable when needed over an extended period, such as in cases of multi-organ involvement [3,23]. In this hazardous clinical context, the immunosuppressants used have included high doses of methylprednisolone, cyclophosphamide, mycophenolate mofetil, methotrexate, azathioprine, rituximab, leflunomide, intravenous immunoglobulins, and cyclosporine [9,34]. Despite the fact that the available evidence is scarce, switching to a biologic non-TNF-related mechanism of action appears to be an appropriate strategy for the subsequent long-term management of IBD. Conversely, if a TNF antagonist is considered essential in IBD treatment, switching to another agent within the same class might be considered in isolated cutaneous LCV, with close follow-up conditions, to identify the relapse of vasculitis early, as this would render the continued use of an anti-TNF agent prohibitive [23]. In Brazil, there are few treatment options available involving biological drugs, especially in public healthcare [35]. In our study, two patients were re-challenged with another anti-TNF agent, and none of them experienced a recurrence of LCV during the follow-up period (a mean of 21.4 months). By contrast, a recurrence rate of 33% was reported in patients who were re-challenged with an alternative anti-TNF agent in a French study [34]. Thus, the decision to re-challenge IBD patients who develop vasculitis secondary to anti-TNF therapy with another TNF antagonist must be made on a case-by-case basis.

The present study had certain methodological limitations that are inherent to retrospective studies. Firstly, there was registration bias, a common feature of retrospective studies. However, in the present study, information on the patients’ clinical and laboratory data and their long-term outcomes was obtained using an electronic database, and the data collection process was standardized by the researchers. These strategies tend to minimize the occurrence of such a bias. 

Secondly, the therapeutic approach to LCV management could have been influenced by the way in which individual clinicians performed the assessments at each center. Nonetheless, the same medication (i.e., prednisone) was used as LCV therapy in all the institutions, albeit in different therapeutic regimens. 

Thirdly, the burden of LCV in the present study may not have completely reflected the true prevalence of LCV in the total population with IBD. In our case series, the IBD patients were from five tertiary IBD referral centers, which meant they were more likely to exhibit more severe IBD symptoms, thus requiring anti-TNF therapy more frequently than patients in a primary care medical setting. This might have biased the results toward a higher prevalence than that found in the general IBD population. Hence, the results should be interpreted with caution. 

Fourthly, as the study population comprised only five patients with anti-TNF-therapy-induced LCV, the results cannot be generalized to the wider population, especially in terms of the optimal therapeutic strategies for use in patients with IBD-associated vasculitis. This extends to questions about the safety of switching from one anti-TNF inhibitor to another (i.e., a departure from the previous treatment). 

In addition, we did not perform direct immunofluorescence to study Ig/immune complex deposition in the biopsy samples. Therefore, we could not completely exclude very rare causes of vasculitis, such as IgA vasculitis, even in the absence of extracutaneous manifestations.

Lastly, LCV secondary to anti-TNF therapy in IBD is a rare condition. Thus, it is unlikely that future studies with prospective longitudinal designs will be carried out to evaluate the nuances of this type of vasculitis in IBD patients.

## 5. Conclusions

LCV is a rare complication resulting from the use of anti-TNF therapy in IBD patients. In this context, clinicians must be aware that an evaluation of IBD patients, especially those in remission who present with unexplained skin rashes during anti-TNF therapy, should include a skin biopsy and a thorough investigation to exclude organ involvement and other conditions associated with vasculitis, such as infections, non-IBD immune-mediated diseases, and malignancies. In these circumstances, it is necessary to consider LCV diagnosis as a paradoxical complication caused by anti-TNF therapy. The complete resolution of cutaneous lesions can be achieved by discontinuing the use of the anti-TNF agent and beginning systemic treatment with steroids.

## Figures and Tables

**Table 1:** Clinical Characteristics of 5 IBD Patients with Leukocytoclastic Vasculitis Associated with TNF Inhibitors.

Patient	Age (y)/Gender	Diagnosis ^a^	IBD Duration (y)	Duration of Treatment (mo) ^b^	IBD Agents	Time to Resolution (Weeks) ^c^	Outcome
1	38/F	CD (A2L3B1)	2	12	IFX	8	Complete remission
2	20/F	CD (A2L3B1)	2	10	ADA	8	Complete remission
3	34/F	CD (A2L1B1)	12	28	ADA + MTX	4	Complete remission
4	32/F	UC (E3)	1	44	ADA	8	Complete remission
5	37/F	UC (E2)	19	60	ADA	12	Complete remission

IBD, inflammatory bowel disease. TNF, tumor necrosis factor. CD, Crohn’s disease. UC, ulcerative colitis. LV, leukocytoclastic vasculitis. IFX, infliximab. ADA, adalimumab. ^a^ According to Montreal classification. In CD patients, A2 means CD diagnosis of between 17 and40 years, L1 means ileal location, L3 means ileocolonic location, and B1 means non-penetrating, non-structuring behavior. In UC patients, E2 means left colitis and E3 means pancolitis. ^b^ Mean duration of anti-TNF therapy. ^c^ Time for complete resolution of skin lesion after anti-TNF withdrawal and initiation of prednisone.

## Data Availability

The datasets used and/or analyzed during the current study are available from the corresponding author on reasonable request.
